# Molecular Psychiatry: Trends and Study Examples

**DOI:** 10.3390/ijms21020459

**Published:** 2020-01-10

**Authors:** Theo Rein, Gabriel R. Fries

**Affiliations:** 1 Translational Psychiatry Program, Department of Psychiatry and Behavioral Sciences, McGovern Medical School, The University of Texas Health Science Center at Houston, Houston, TX 77054, USA; 2 Department of Translational Science in Psychiatry, Max Planck Institute of Psychiatry, 80804 Munich, Germany

In contrast to about 20–30 years ago, the concept that psychiatric diseases have a molecular basis is now widely accepted. Nevertheless, the complexity of these diseases poses a particular challenge for the translational efforts to integrate disease manifestation, behavior, neuronal circuits, and molecular pathways to form a complete theory with significant clinical implications. The Special Issue “Molecular Psychiatry” of the International Journal of Molecular Sciences presents exciting examples of these efforts (9 reviews and 11 original articles), encompassing studies on molecular mechanisms, animal models, biomarkers, advanced methodology, drug development and responsiveness, as well as genetics and epigenetics. 

## 1. Molecular Mechanisms

Basic science mechanisms are a vital part of the search for the biological basis of psychiatric disorders, providing molecular hints that can later be tested as biomarkers or as targets for the development of new medications. Several manuscripts published in this Special Issue describe interesting mechanisms that may underlie the biology of these disorders.

The role of corticosteroid receptors in psychiatric diseases has been recognized for a long time, in part as executors of the stress response that is pivotal in a number of diseases. The review of Baker et al. illustrates this while focusing on the molecular mechanisms regulating steroid receptor activity [1]. The authors summarize our current knowledge on the control of glucocorticoid receptor (GR) activity by the heat shock protein (Hsp) 90 based chaperone system, with a focus on the established stress factor and co-chaperone FK506 binding protein (FKBP) 51. The link to the stress response and circadian rhythm is outlined and the potential for chaperone-targeting therapeutics is discussed. 

The article by Kretzschmar and colleagues focusses on the molecular effects of the stress- and GR-inducible protein Downregulated in renal cell carcinoma 1 (DRR1) in organizing the actin cytoskeleton [2]. DRR1 has been associated with several brain disorders and is described as a resilience factor. The authors demonstrate that DRR1 affects actin dynamics through several mechanisms that likely impact neuronal function, as well as stress physiology and pathophysiology. 

The article by van Weert et al. provides intriguing mechanistic insight into the regulation of genes that can be target of either GR or the mineralocorticoid receptor (MR) [3]. It is established for a long time that the balanced activity of these two corticosteroid receptors is pivotal for stress coping and mental health. The authors provide evidence suggesting that the basic helix-loop-helix transcription factor NeuroD facilitates MR binding to gene regulatory elements, questioning competition for DNA binding as a mechanism of MR- over GR-specific binding.

Strategies to decode the molecular mechanisms of fast-acting antidepressants are the focus of the review by Herzog and colleagues, with emphasis on gender-specific aspects [4]. The authors survey the literature documenting the need for elucidating gender-specific mechanisms and provide the current state of the art to propose a framework for experiments in rodents to tackle the issue of gender difference in treatment response. 

In the search for molecular mechanisms of the protein Disrupted in Schizophrenia 1 (DISC1), whose gene translocation frequently is found correlated with cases of schizophrenia, bipolar disorder, and major depression, Ramos et al. followed an unbiased proteomic approach [5]. The analysis of the proteome of primary neurons in which DISC1 was knocked down provided evidence that DISC1 has a role in both neurodevelopment and synaptic function.

In light of the accumulating evidence for the association between chronic inflammation and major depressive disorder (MDD), Milenkovic et al. review the role of chemokines in MDD. Chemokines are known as small cytokines impacting the induction of chemotaxis, the migration of leukocytes and macrophages, and the propagation of inflammation. The authors conclude that these cytokines could serve as peripheral markers of psychiatric disorders, or even targets for novel treatment strategies in depression [6].

Finally, the article by Ambrée et al. examining mechanistic aspects of the T cell response in stress-induced depression-like behavior [7] is outlined in the next section on animal models in more detail.

## 2. Animal Models

The application of basic science findings to the clinics is an important and particularly hard part of translational science. One of the commonly used approaches to aid in this bench-to-bed translation is the use and study of so-called ‘animal models’ that can test specific mechanisms in vivo prior to the use of human subjects. This Special Issue includes two interesting articles applying such models.

Ambrée and colleagues used social defeat stress (SDS) as a model for stress-induced depression-like behavior to shed light on the T cell phenotype associated with susceptibility and resilience to depression [7]. The authors grouped SDS-exposed mice into susceptible and resilient and found significantly increased numbers of interleukin-17 producing CD4+ and CD8+ T cells in the spleen of susceptible mice. Harnessing the power of genetic intervention, mice with a conditional deletion of PPARγ in CD4+ cells were analyzed; PPARγ is an inhibitor of Th17 development and its deletion thus enhances Th17 differentiation. However, this genetic manipulation did not change susceptibility to SDS. Thus, while SDS promotes Th17 cell and suppresses Treg cell differentiation predominantly in susceptible mice, the effects in immune responses after stress exposure remain to be elucidated [7].

Another rodent model for susceptibility and resilience to stress-induced depression-like behavior is represented by the Roman High-Avoidance (RHA) and the Roman Low-Avoidance (RLA) rats, which were used by Serra et al. to investigate the effects of forced swimming on factors of neuronal plasticity [8]. This stressor elicited changes in the expression of brain-derived neurotrophic factor (BDNF), its receptor trkB, and the Polysialilated-Neural Cell Adhesion Molecule in distinct regions of the brain. These changes pronouncedly differed between the two rat lines, consistent with a role of BDNF/trkB signaling and neuroplasticity in susceptibility and resistance to stress-induced depression [8]. Animal models are also outlined in the above-mentioned review, where Herzog et al. suggest procedures for experiments in rodents to investigate gender differences in treatment response in depression [4]. 

## 3. Biomarkers

As is the case in other medical fields, such as oncology and cardiology, biomarkers may provide valuable proxy information in psychiatric patients, potentially assisting in measures of prognosis, treatment response, diagnosis, and progression. Psychiatric disorders pose a particular challenge due to the tissue specificity of many currently investigated biomarkers; i.e., not all blood-based measures directly represent changes in the brain, and the study of the correlations between the periphery and the central nervous system is a rapidly evolving field. This Special Issue includes five manuscripts focused on the challenges of identifying clinically and biologically relevant biomarkers for psychiatric disorders.

Recent findings in the field of Psychiatry have suggested a potentially key role for epigenetic mechanisms in determining not only risk and resilience in patients and vulnerable subjects but also in modulating one’s response to a given treatment. The paper by Goud Alladi and colleagues explored through a systematic review the evidence of DNA methylation mechanisms involved in the clinical treatment response in serious mental illness, specifically bipolar disorder, schizophrenia, and major depressive disorder [9]. The authors of this interesting study emphasize the potential clinical use of such markers in predicting whether a patient will respond or not to a medication, which is a highly anticipated approach in the emerging field of personalized medicine.

Among promising biomarkers, significant effort has also been made in the study of inflammatory mediators to predict diagnosis, prognosis, and treatment. In fact, most psychiatric disorders have been shown to present immune dysfunctions, as measured by cytokines and inflammatory mediators, and such molecular phenotypes are thought to at least partly mediate the higher cardiovascular and metabolic disturbances seen in psychiatric patients. Baghai and colleagues, for instance, performed a large study to investigate the combined influence of major depressive disorder and cardiovascular disorders on immune mediators [10]. Interestingly, their findings not only suggest that higher inflammatory biomarkers underlie the risk for cardiovascular disease in depression patients but also that higher levels of these molecules are associated with better clinical outcome and faster remission in patients. 

This Special Issue also includes the study by Mühle and colleagues [11], which investigated another type of peripheral biomarkers in alcohol-dependent patients: the acid sphingomyelinase, an enzyme that breaks sphingomyelin into ceramide and thereby changes the composition of plasma membranes. As hypothesized, the authors found clinically relevant changes in the enzyme levels in patients, which also correlated with several other biochemical markers of dependence and health. 

Another interesting approach has been taken by Rotter and colleagues [12], which measured the expression levels of alpha-synuclein (a protein known to be associated with Lewy bodies in Parkinson’s disease) in the peripheral blood of patients with major depressive disorder as an attempt to understand the high comorbidity between depression and Parkinson’s disease. Accordingly, their interesting findings were suggestive of an increase in alpha-synuclein levels in depressed patients, with a positive correlation between the severity of depression and the levels of this biomarker. 

Finally, Steiger and Pawlowski discussed in a review paper how biomarkers do not necessarily entail laboratory measures using biospecimens [13]. The authors discuss the use of sleep electroencephalogram (EEG) as a biomarker of impaired sleep in the context of depression, and comprehensively review many applications and biological underpinning of this marker in the depressed population. 

## 4. Advanced Methods

One of the challenges of the field of molecular psychiatry is related to methodological limitations. Many commonly used techniques are particularly limited and have not been successful in answering the rapidly evolving questions of the field. Two manuscripts in this Special Issue are focused on innovative approaches that can revolutionize the field, including the use and proper analysis of Big Data through machine learning methods and the development and application of induced pluripotent stem cells (iPSC) from psychiatric patients.

The study by Cao and colleagues tested eight machine learning algorithms in the same transcriptome-wide expression datasets of patients with schizophrenia and controls to identify reproducible biological signatures of disease [14]. This type of study to identify the most robust and effective algorithms can have significant impacts in the field since integrative analysis of complex datasets is becoming the cornerstone of biological psychiatry studies.

Novel methods are also being developed at the bench side, including the establishment of patient-derived iPSCs. These cells can provide an effective tool for the study of complex diseases by allowing the establishment of cellular models accounting for the patient’s genetic background while removing the effect of outside environmental influences. The review by Hoffman and colleagues [15] provides an insightful overview of the use of these cell models to study the neurobiological basis of childhood-onset schizophrenia, discussing advantages, limitations, and challenges of the field and the use of these modern technologies. Overall, the discussion also applies to other psychiatric diagnoses which may benefit from the use of patient-derived cell models as opposed to commonly used animal primary cells or cell lines. 

## 5. Drugs/Antidepressant Response

One of the major limitations of currently available psychiatric medications stems from the long duration of treatment and the need for several days of treatment until the detection of proper medication response and efficacy. Due to the heterogeneity of patients and the lack of proper biomarkers of treatment response, more often than not the choice of treatment is made in a trial-and-error mode by the clinician, potentially delaying symptom resolution. Several authors aim at understanding the specific mechanisms of actions of existing drugs with the ultimate goal of identifying ways to predict treatment response in patients. Three papers in this Special Issue provide an overview of this area of investigation, with a particular focus on major depression and schizophrenia.

The abovementioned review by Herzog et al. focusses on gender-specific aspects of antidepressant response [4]. The article by Ising and colleagues presents the analysis of FKBP5 gene polymorphism and of RNA and protein levels of its gene product FKBP51 in peripheral blood in 297 inpatients treated for acute depression according to doctor’s choice [16]. Pronounced reduction of FKBP5 gene and FKBP51 protein expression was observed in patients responding to antidepressant treatment, while non-responders had increased levels. The FKBP5 genotype moderated this effect [16]. This study significantly contributes to the complexity of the link of the stress factor FKBP51 to antidepressant responsiveness.

The review by Kondej and colleagues discusses established and novel drug targets for schizophrenia, in particular, the concept of multi-target drugs [17]. This is an increasingly important aspect in drug discovery, also considering the limited success of single-target drugs in polygenic diseases with complex pathomechanisms. 

## 6. Genetics and Epigenetics

Family studies have convincingly proven that psychiatric disorders run in families and have a strong genetic basis. Notwithstanding, the high heterogeneity of psychiatric diagnoses has significantly hindered the discovery of their molecular genetics, with few genome-wide association studies (GWAS) suggesting the need for extremely large samples and robust statistical methods. In addition, the ‘heritability gap’ (i.e., the gap between the heritability detected in family studies and that detected by GWAS) has suggested an important role for the environment in modulating genetic mechanisms, which has led to the hypothesis that epigenetic mechanisms may be especially important in these disorders. Three manuscripts in this Special Issue go deeper into this topic and highlight approaches and findings related to the genetics and epigenetics of psychiatric disorders.

Lesiewska and colleagues undertook an experiment to investigate the association between affective temperament traits and polymorphisms in dopaminergic genes in a sample of obese subjects [18]. Among other findings, they found interesting associations between specific temperament dimensions with a polymorphism in the catechol-O-methyltransferase (*COMT*) gene, which supports the hypothesis of a key role for dopaminergic genes in determining temperament expression in obese individuals. 

The influence of genetic markers in psychiatric traits is also subject of the review from Ohi and colleagues [19], which explored the genetic determinants of general cognitive function and how they overlap with schizophrenia. Lower general cognitive function has been repeatedly associated with a higher risk for schizophrenia, and a genetic risk may likely underlie this association. More specifically, the authors discussed risk *loci* identified by GWAS studies of both schizophrenia and cognitive function, the polygenic nature of both conditions, and recent evidence showing how their genetic determinants are particularly similar. 

Finally, in light of evidence suggesting an important role for the serotonergic system in many physiological and pathological conditions, including psychiatric disorders, the paper by Rebholz and colleagues reviewed biological findings regarding the serotonin 4 receptor (5-HT4R) [20] in brain regions and how its genetic regulation and gene expression changes can modulate reward and executive function and potentially give rise to mood changes in vulnerable subjects. The authors also extend their discussion to explore methodological advancements that will be needed for a better understanding of this receptor in brain function, including the development of more suitable genetic mouse models. 

Overall, this rich collection of studies provides a diversified portfolio of the several approaches that can be used for tackling the biology of psychiatric disorders, reinforcing the importance of using multiple lines of converging evidence for their study. While not exhaustive and comprehensive, these studies are effective in identifying current limitations of the field and provide the reader with an enlightening overview of the directions and future of translational research in molecular psychiatry.
